# Modulation of cough response by sensory inputs from the nose - role of trigeminal TRPA1 versus TRPM8 channels

**DOI:** 10.1186/1745-9974-8-11

**Published:** 2012-12-03

**Authors:** Tomas Buday, Mariana Brozmanova, Zuzana Biringerova, Silvia Gavliakova, Ivan Poliacek, Vladimir Calkovsky, Manjunath V Shetthalli, Jana Plevkova

**Affiliations:** 1 Department of Pathophysiology, Comenius University, Jessenius Faculty of Medicine Martin, Sklabinska Str. 26, Martin, 036 01, Slovak Republic; 2 Clinic of Anaesthesiology and Emergency Medicine, Jessenius Faculty of Medicine and University Hospital, Martin, Slovak Republic; 3 Department of Medical Biophysics, Comenius University, Jessenius Faculty of Medicine, Martin, Slovak Republic; 4 Clinic of Ear Nose throat Diseases, Head and Neck Surgery, Jessenius Faculty of Medicine and University Hospital, Martin, Slovak Republic; 5 Pediatric Department, Royal Gwent Hospital, Newport, Wales, United Kingdom

**Keywords:** Cough, Nose, Irritants, Menthol, Trigeminal, Olfactory

## Abstract

**Background:**

Cough, the most important airways defensive mechanism is modulated by many afferent inputs either from respiratory tussigenic areas, but also by afferent drive from other organs. In animal models, modulation of cough by nasal afferent inputs can either facilitate or inhibit the cough response, depending on the type of trigeminal afferents stimulated.

**Methods:**

In this study we addressed the question of possible bidirectional modulation of cough response in human healthy volunteers by nasal challenges with TRPA1 and TRPM8 agonists respectively. After nasal challenges with isocyanate (AITC), cinnamaldehyde, (−) menthol and (+) menthol (all 10^-3^ M) nasal symptom score, cough threshold (C2), urge to cough (Cu) and cumulative cough response were measured).

**Results:**

Nasal challenges with TRPA1 relevant agonists induced considerable nasal symptoms, significantly enhanced urge to cough (p<0.05) but no statistically significant modulation of the C2 and cumulative cough response. In contrast, both TRPM8 agonists administered to the nose significantly modulated all parameters including C2 (p<0.05), Cu (p<0.01) and cumulative cough response (p <0.01) documenting strong anti irritating potential of menthol isomers.

**Conclusions:**

In addition to trigeminal afferents expressing TRP channels, olfactory nerve endings, trigemino – olfactoric relationships, the smell perception process and other supramedullar influences should be considered as potential modulators of the cough response in humans.

## Background

The cough reflex is not a static phenomenon, but is a flexible entity modulated by many central and peripheral neuronal mechanisms, sometimes termed ‘cough plasticity’ [1]. Stimulation of trigeminal terminals in the nose by the TRPV1 agonist capsaicin, and histamine significantly enhances cough response induced in laboratory animals. Such stimulation also up regulates cough responsiveness in human healthy subjects and patients with allergic rhinitis [2-5]. These findings suggest cough is enhanced by increased afferent drive form the nose to the sensory trigeminal nuclei and then by cooperation with the brainstem neuronal circuits modulating cough. Nucleus tractus solitarius (nTS) is believed to be the site important for modulation of cough reflex at the central level [6]. Naturally, if noxious substance enter the airways through the nose, then defensive mechanisms in this region should be up regulated and prepared for airway protection.

Recently we studied the mechanism underlying the antitussive action of menthol, which is frequently used in many over–the–counter preparations for cold and cough treatments, the majority of which are made for nasal application (drops, sprays). Despite their frequent use and recognised antitussive action [7,8] the exact mechanism of menthol action had not been elucidated. Based on a series of studies performed on conscious and anaesthetized guinea pigs, and using single cell RT PCR method we suppose that antitussive action of menthol is mediated by nasal trigeminal afferents, more accurately by subpopulation of TRPV1-/TRPM8+ expressing neurons [9]. We are aware of species differences, but guinea pig is an ideal model to study cough because the neurophysiology and neuropharmacology of airway afferents are very similar to the human condition.

Taken together, cough seems to be both up regulated and down regulated by distinct populations of nasal afferents. Signalling from nociceptors may primarily up regulate cough, whereas activation of other nasal nociceptors may down regulate it. To test this hypothesis we employed nasal nociceptive stimulation by TRPA1 ligands and nasal menthol challenges to observe whether and how it does influence the cough responsiveness in humans. Nasal nociceptive stimulation was performed by TRPA1 agonists allyl-isothiocyanate (AITC) and cinnamaldehyde which actually may represent a model for air-born irritants induced signalling [10]*.* The broad spectrum of irritants like acrolein, isocyanates, oxidizing substances and other pollutants activate the cation channel TRPA1 by covalent modification of the channel protein [11,12]. Activation of this channel leads to a sensation of airway irritation [13]. TRPA1 agonists have been identified as potent tussive stimuli in animal models and humans and provide a promising evidence to suggest TRPA1 as a relevant antitussive targets with possible clinical applications [14,15]. Nasal trigeminal TRPA1 as well as TRPM8 stimulation had never been studied regarding modulation of the cough reflex in humans.

### Aim of the study

The aim of the present study was to assess the cough responsiveness in healthy human volunteers after intranasal challenges of TRPA1 agonists AITC and cinammaldehyde and TRPM8 agonists (−) menthol and (+) menthol.

## Methods

### Study population

The study population consisted of 20 volunteers for TRPA1 relevant challenges and 18 volunteers for TRPM8 relevant challenges (equal gender distribution, average age 23 yrs, non-smokers, non atopic, with normal lung functions tests, anterior rhinoscopy, and without acute respiratory infection in preceding 4 weeks). Based on structured interviewer-led questionnaire, each subject was asked about respiratory symptoms and his/her individual and family history of bronchial asthma, allergic rhinitis, gastroesophageal reflux, cardiovascular diseases and ACE inhibitor treatment. After review of lung function tests, ear-nose-throat examination and questionnaire, the subjects were considered as meeting (or not) inclusion criteria for the study.

The study was approved by the Committee for Ethics of the Jessenius Faculty of Medicine in Martin. Informed consent was obtained from all study subjects. However, the information on study endpoints given to subjects was limited to minimise potential conscious influence by participants on cough and urge to cough.

### Assessment of cough sensitivity (CS)

Cough sensitivity testing was performed according to ERS Cough Taskforce recommendations [16]. Capsaicin (30.5 mg) was dissolved in 1 ml of pure ethanol and 1 ml of polyoxyethylene sorbitan (Tween 80) and then further dissolved in 8 ml of normal saline to yield a 0.01 M stock solution. The solution was diluted with saline in order to obtain serial doubling concentrations ranging 0.49–1,000 μM all freshly made the day of testing. In order to increase cough challenge blindness, inhalations of saline (placebo) was randomly interspersed between incremental concentrations of capsaicin. This strategy is used to reduce the effects of voluntary suppression or conditioned responses in subjects who would otherwise be anticipating progressively higher concentrations of tussive agent [16]. Each subject tested had performed lung function tests and inhalation of saline aerosol before increasing concentrations of aerosols of capsaicin solutions were applied. Aerosols were produced by computer assisted compressed air driven nebulizer Koko Digi Doser with controlled nebulizer output per breath. The lowest concentrations of capsaicin induced two or more coughs (C2) and five or more coughs (C5) were determined, log transformed and the dose response curves of the total number of coughs were constructed. According to the ERS recommendations, only coughs that appeared immediately after aerosol inhalation had been counted. Subjects were simultaneously asked to subjectively evaluate the urge to cough based on their “perceived” airway irritation after capsaicin inhalation until they reached C2.

### Assessment of urge to cough

Urge to cough is defined as the sensation of airway irritation that precedes the motor act of cough. This urge to cough phenomenon represents the cortical neural contribution to the airway defence system [17]*.* However, it is difficult to measure it accurately, because it is an exclusively subjective phenomenon. We used the method described by Dicpinigaitis et al. [18], we estimated Cu - the first concentration of capsaicin aerosol which induced urge to cough, and we asked our subjects to evaluate and compare verbally the feelings regarding the urge to cough after each capsaicin inhalation.

### Nasal challenges

The subject^′^s head was tilted carefully on one side and using a micropipette 20 μl of the particular agonists or (saline)/vehicle, respectively, had been applied into the nostril of the contralateral side, approximately 1.5 – 2 cm from the external orifice. The same manner was used for application of agonists into contralateral nostril. The external surface of the nose was gently massaged for a few seconds to spread instilled volume on a larger part of the nasal mucosa. 1% DMSO was used as the universal vehicle for all of delivered apolar substances. Stock solutions of AITC (10^-1^ M, Sigma) and cinnamaldehyde (10^-1^ M, Sigma) in 1% DMSO, had been further diluted in saline (to 10^-3^ M) every day prior the challenges. (+) menthol and (−) menthol (both Sigma) as polar alcohols, were diluted in ethanol to stock (10^-1^ M) and further diluted in saline (to 10^-3^ M) at the day of the challenges, as well. It is known that ethanol may potentiate cough reflex as it was documented in guinea pigs and humans already, but in our study final concentration of ethanol applied onto the nasal mucosa was 0.5%.

### Nasal symptoms

We have used previously modified scoring system to assess intensity of nasal symptoms. However in present protocols subjects never sneezed after nasal challenges, they only reported burning and unpleasant olfactory sensation after nasal AITC, slight burning only after cinnamaldehyde. Intranasal menthol challenges did not induce subjective feeling that may be described as “symptoms” or unpleasant sensations, only cooling. Therefore we decided to evaluate burning sensations using modified scale from 0 to 10 (10 is the most intensive burning the subject has ever experienced). Cooling sensation after menthol challenges was determined similarly. The amount of nasal secretions was also assessed by means of weighing of used handkerchiefs (only for AITC challenge, as other challenges did not provoke considerable rhinorrhoea).

### Data analysis

Capsaicin cough thresholds C2, C5, and urge to cough are expressed as geometric mean with 95% confidence interval. The total numbers of coughs for the dose response curves are presented as mean and standard error of mean. For the statistical analysis repeated measures ANOVA and Friedman’s test as appropriate were employed using statistical programme GraphPad InStat. The differences were considered significant at p < 0.05.

### Study protocol

Subjects took nasal challenges with subsequent cough sensitivity testing once a week same time of the day. Prior to this, they underwent general medical examination, lung function testing, and ear-nose-throat examination. Cough sensitivity was determined in volunteers after nasal saline challenge first, and later after intranasal vehicle, intranasal AITC (10^-3^ M) cinnamaldehyde (10^-3^ M) for TRPA1 arm of the study. The same pattern was applied for TRPM8 arm of the study; capsaicin cough tests were performed after nasal saline, nasal vehicle, (−) menthol (10^-3^ M) and (+) menthol (10^-3^ M) (dissolved in ethanol and further diluted in saline). Challenges were performed in randomized order, except the first one challenge after nasal saline, which was fixed. Randomization was used to minimize the startle reaction which has been described in humans during repeated cough challenges.

## Results

### Nasal symptoms

**Figure 1:**
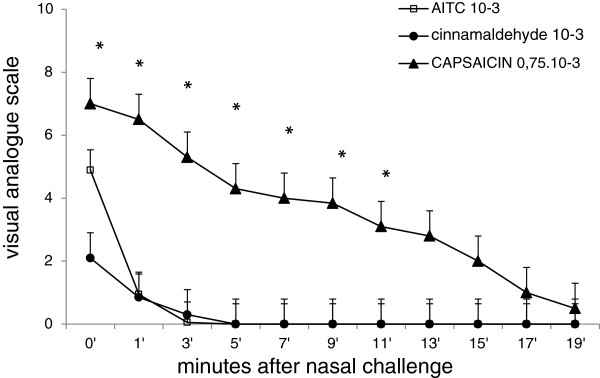
**Represents intensity of burning sensation induced by intranasal administration of AITC, and cinnamaldehyde (10**^**-3 **^**M) in present study in comparison to burning induced by intranasal capsaicin 0.75 10**^**-3**^**M (Plevkova et al.,**[5]**).** As it could be seen from the trace, TRPA1 agonists are significantly less effective when comparing to TRPV1 agonist capsaicin. Thus we speculated that less effective trigeminal drive was not enough to modulate cough response in our set up, *p<0.05.

Administration of isocyanate and cinnamaldehyde induced burning sensation when topically applied onto the nasal mucosa. Administration of isocyanate also induced lacrimation and eye irritation. Subjects described strong irritation and burning accompanied by horseradish or wasabi smell immediately after the challenge. However, this burning suddenly and rapidly minimized, while application of even lower concentrations of capsaicin [3] induced burning with the intensity 7–8 at 10 point scale lasting for more than 20 minutes (Figure 1). Application of cinnamaldehyde induced only negligible burning and subjects reported the smell sensation of cinnamon to be more intense then burning. Application of (−) and (+) menthol induced no burning, but a cooling sensation in all subjects.

Regarding other nasal symptoms, only nasal isocyanate induced measurable discharge (secretion), but other stimuli failed to induce any considerable symptoms including nasal mucosal swelling or discharge.

Similarly, all nasal challenges induced no significant changes in airway expiratory flows (nasobronchial reflex – narrowing of airway lumen by nasal stimulation). No considerable drop of FEV_1_ or FVC was detected after nasal TRPA1 and TRPM8 relevant challenges (Table 1).

**Table 1:** Values of FEV1/FVC after intranasal challenges with saline, vehicles and both TRPA1 and TRPM8 agonists expressed as % of predicted

**TRPA1 line**	**Saline**	**Vehicle**	**AITC**	**Cinnamaldehyde**
mean ± SD	99.2 ± 0.06%	97.7% ± 0.07	98.4% ± 0.06	98.5% ± 0.06
**TRPM8 line**	**saline**	**vehicle**	**(+) menthol**	**(−) menthol**
mean ± SD	99.6% ± 0.08	98.1% ± 0.09	98.4% ± 0.06	99.8% ± 0.06

Note: data are expressed as % of predicted values.

### Cough reflex sensitivity and cumulative cough count for TRPA1 line

**Figure 2:**
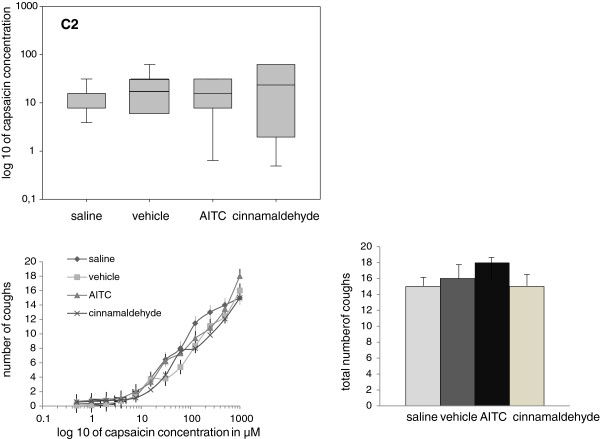
**Upper panel represents the changes of cough threshold - parameter C2.** As it could be seen C2 values are not significantly different when comparing data obtained after nasal saline, vehicle, AITC and cinnamaledyde. Cumulative and total cough response obtained during the cough tests after nasal AITC and cinnamaldehyde do not differ from saline and vehicle values (lower panel) (all p>0.05).

Data obtained for C2 and C5 were shifted from baseline and vehicle values, however, difference did not reach statistical significance. The only parameter which was significantly influenced was urge-to-cough after intranasal isocyanate challenge (Table 2 and Figure 2).

**Table 2:** Values of cough reflex sensitivity obtained in TRPA1 line of the study

	**Saline**	**Vehicle**	**Isocyanate**	**Cinnamaldehyde**
**C2**				
**Cu**	10.1	13.8	6.8 *	12.1
**C2 GM**	12.5	16.8	10.8	12.6
**GSD**	3.2	2.6	4.3	6.7
**CI min**	8.3	10.6	6.8	7.5
**CI max**	15.7	17.2	12.8	13.7
**C5**				
**GM**	198.4	183.7	277.4	287.1
**GSD**	4.4	4.8	4.3	3.46
**CI min**	196.4	181.6	148.4	166.5
**CI max**	200.4	185.8	518.4	495.36

Note: data are expressed in μM, GM – geometric mean, GSD – geometric standard deviation, CI min, CI max – 95% confidence intervals with alpha = 0.05. *p<0.05, Cu – urge to cough, C2 – concentration of capsaicin inducing 2 or more coughs, C5 - concentration of capsaicin inducing 5 or more coughs.

Cumulative cough response obtained during the cough tests after nasal isocyanate and cinnamaldehyde challenges do not differ significantly from baseline and vehicle values (Figure 2). The total number of coughs obtained during one cough test after nasal saline, vehicle, isocyanate and cinnamaldehyde are (15.2 vs 16.1 vs 18.3 vs 15.4 coughs) respectively (all p>0.05). Also regression analyses did not show significant differences.

### Cough reflex sensitivity and cumulative cough count for TRPM8 line

**Figure 3:**
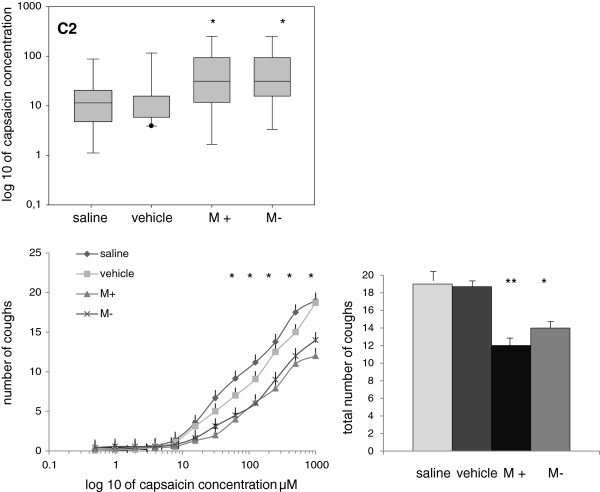
**Menthol nasal challenges decreased cough sensitivity, what is demonstrated by the higher capsaicin concentrations for the cough threshold C2 (upper panel) compared to the data obtained after nasal vehicle and saline data (both isomers p<0.05 for C2).** Cumulative cough counts and total cough response after nasal (+) and (−) menthol challenges decreased significantly compared to the saline and vehicle values p< 0.01. Regression analysis showed similar differences *p<0.05, **p<0.01.

Menthol nasal challenges decreased cough sensitivity demonstrated by higher C2 capsaicin concentrations (Table 3; Figure 3) compared to the baseline and vehicle data (both p<0.05).

**Table 3:** Values of cough reflex sensitivity obtained in TRPM8 line of the study

	**Saline**	**Vehicle**	**(+) Menthol**	**(−) Menthol**
***C2***				
**Cu**	11.8	12.7	26.5 *	22.4*
**C2 GM**	12.5	14.7	31.3 *	26.3*
**GSD**	4.6	2.7	5.9	6.1
**CI min**	10.4	11.3	28.9	23.8
**CI max**	14.7	17.2	33.6	28.7
**C5**				
**GM**	261	249	458.5	478.8
**GSD**	4	4.3	3.1	2.9
**CI min**	259	248	456.8	477
**CI max**	263	251	460.2	480.5

Note: data are expressed in μM, GM – geometric mean, GSD – geometric standard deviation, CI min, CI max – 95% confidence intervals with alpha = 0.05. *p<0.05, Cu – urge to cough, C2 – concentration of capsaicin inducing 2 or more coughs, C5 concentration of capsaicin inducing 5 or more coughs.

Cumulative cough counts after nasal (+) and (−) menthol challenges (12.2 and 14.1 coughs, respectively) were lower compared to the saline and vehicle values (19.1 and 18.7 coughs; both p < 0.01). Regression analysis showed similar differences.

### Assessment of urge to cough and subjective sensations after nasal challenges

The lowest concentration of capsaicin that induced an urge to cough was estimated based on study participants’ subjective evaluation during the capsaicin test after particular nasal challenges. Nasal TRPA1 agonist isocyanate challenge induced a “very unpleasant” sensation, bringing negative emotional assessment to this challenge. Otherwise cinnamaldehyde nasal challenge was evaluated by most subjects as being “not bad” with mainly olfactory sensation of cinnamon. No adverse reaction but rather a very short mild burning of nasal mucosa at the moment of the challenge was generally reported. In two subjects nasal cinnamaldehyde induced attack of migraine 1 hour after the challenges. Urge to cough after nasal AITC occurred at lower capsaicin concentrations (Figure 2, Table 2) and subjects reported subjectively considerable airway irritation. Urge to cough after cinnamaldehyde was not influenced significantly (Table 2).

Menthol nasal challenges produced in subjects refreshing feeling and cooling sensation. Similarly to the cough threshold, urge to cough was shifted to higher capsaicin concentrations. Subjects reported that menthol in the nose made capsaicin airway irritation less intense, and subjectively they tolerated the capsaicin test better (Table 3, Figure 3).

## Discussion

Nasal challenges with (+) and (−) menthol (TRPM8 agonists) have beneficial effects in suppression of subjectively evaluated airway irritation, increasing capsaicin cough threshold and in inhibition of the cough response. On the other hand expected enhancement of cough due to activation of TRPA1 by nasal isocyanate and cinnamaldehyde was not observed.

This study assessed the modulation of cough response induced by nasal stimulation of TRPA1 and TRPM8 channels. TRPA1 is the channel most likely stimulated by air born pollutants, oxidizing substances, and endogenous inflammatory products and is categorized as nociceptive. It is responsible for induction of burning, irritation and pain [12]. TRPM8 channel is mainly thermoreceptor with activation threshold around 24°C, giving the feeling of innocuous cold and freshness. It could also be activated by menthol [13,14] which is known to have contra irritating effects in the airways [15]*.*

Nasal menthol challenges have an antitussive effect in awake and anaesthetized guinea pigs [9]. The main targets for menthol action in the airways are the trigeminal afferents which are distributed widely in the nasal mucosa. More than 60% or nasal trigeminal afferents express TRPM8 channel, some of those afferents are TRPV1-/TRPM8+ [9] and expression of TRPM8 in nodose and jugular neurons innervating lower airways was less frequent. We propose, that these terminals, which are not nociceptors (as are TRPV1-) may be involved in the down regulating cough observed by nasal menthol application in guinea pigs. Animal models for cough studies and the results obtained in guinea pig models cannot be conclusively applied to the clinical conditions. Although the guinea pig vagus nerve pharmacology and physiology is very similar to human vagus nerve, these results should always be considered and interpreted with caveats [16]. In the present study in human volunteers, menthol was delivered only to the nose (using a micropipette). It is believed that this nasal challenge [3,19] minimizes leaking of menthol deeper into the airways. Although the menthol is highly volatile substance, the possibility for further movement of menthol vapours could not be completely excluded. Further we would suggest that these minimal amounts of menthol (if present) in lower airways would be insufficient to modulate cough reflex.

Studies estimating the effect of menthol on cough reflex in humans are conflicting. Morice and colleages reported antitussive effect of menthol vapours after oral inhalation [7]. However, recent studies document no effect of inhaled (−) menthol on the cough reflex in children [20] or patients prior bronchoscopy [21]. We think that effect of menthol in our study and also in the study by Morice et al. is local, influencing airway afferents involved in modulation of cough reflex.

It is also possibile that systemic exposure of lung and airway afferents to the menthol reabsorbed from nasal musoca is responsible for the observed effect. It was identified that TRPM8 nerve fibres are abundant in nasal mucosa particularly around blood vessels, and may mediate neurovascular reflexes [22]. Johnson et al. in 2009 [23] reported that menthol is involved in regulation of vascular tone, and it may be responsible for vasodilatation. This mechanism together with the abundance of nasal mucosal vasculature may be responsible for systemic absorption of menthol with subsequent influence on lung and airway afferents. However we did not measure the serum concentration of menthol after administration of nasal drops. A study of transdermal absorption and bioavailability of menthol after dermal application of patch by Martin & Valdes, reported detectable but very low plasma concentrations of menthol. As menthol has an antitussive effect after oral administration in guinea pigs in a dose 100 mg/kg of body weight (Brendan Canning, unpublished data), vapors entering the nose after oral administration and the systemic effects need to be considered.

Our controlled study shows that nasal menthol challenge in humans suppresses cough reflex induced by inhalation of capsaicin. Moreover, menthol can stimulate not only trigeminal but also olfactory terminals and activate cortical voluntary cough related pathways. This complex action of menthol brings cooling and freshness sensations, and reduces dyspnoea and respiratory drive perception [24] possibly with typical mint smell. Menthol also decreases trigeminal sensitivity to irritants [25]. Both molecules of menthol isomers (+) and (−) were effective equally, but subjects tolerated (+) menthol challenge better. Subjects were blinded to the substances (+ and – menthol) during the challenge, however, questioning them after the testing they considered (+) menthol more pleasant compared to (−) menthol, which mimicked over the counter drug smell.

Cough is modulated by the cortical influences [26] and many other factors including behavioural, attentional or cognitive may also modulate cough response [27]. We speculate that subjectively pleasant sensations may inhibit the urge to cough by the cortical mechanism, whereas unpleasant sensations may enhance it.

We expected that nasal challenges with TRPA1 agonists would enhance cough response similarly as it does TRPV1 agonist capsaicin challenge in animals and humans [2,3]. Obviously, our data show that cough modulation via nasal TRPA1 is less intensive than that after TRPV1 agonist challenge. Activation of TRPA1 induces symptoms (e.g. burning) to 1/3 of those induced by equal concentration of agonists for TRPV1 (Figure 1). Afferent nociceptive drive induced by nasal capsaicin challenge was 7/10 on the VAS and considerable burning lasted up to 15–20 min. In present subjects (with nasal TRPA1 agonists) burning or unpleasant sensations reached 5/10 and disappeared after 2–3 minutes. It is possible that higher concentrations of isocyanate or cinnamaldehyde, repeated challenges, or challenges in subjects with trigeminal sensorineural hyperresponsiveness might modulate cough parameters. It had been documented that TRPA1 agonists induce cough in guinea pigs and humans after they are inhaled into the lower airways [14]. The C2 concentration was around 200 mM of cinammaldehyde. For nasal challenges in mice 1% toluene diisocyanate had been used by Taylor Clark et al. [28]. All of these studies proved a strong irritation effect of TRPA1 activating substances. However, naturally present irritants, either indoor, or outdoor, are present in inhaled air in very low concentrations. Thus, we used the lowest concentrations capable to induce sensory activation to mimic situation of natural exposure to air born irritants. In pre tests we identified appropriate concentrations for given agonists at 10^-3^ M, which was quite well tolerated, but induced nasal symptoms.

In addition, nasal challenge with isocyanate in our experimental set up modulated the urge to cough. We assume that perception of airway irritation induced by nasal AITC challenge could interfere with the irritation induced by the inhalation of capsaicin itself during the cough test. Subjects already reached the concentration for urge to cough, so they were conscious of the airway irritation and unpleasant sensations possibly leading to cough long before they reached the C2. Early presence of urge to cough during the capsaicin test after nasal isocyanate documented the nociceptive capabilities of nasal TRPA1 agonists by possibly enhancing afferent drive induced by the capsaicin inhalation. In contrast, nasal TRPM8 agonists reduced the urge to cough. After menthol nasal challenge subjects tolerated the capsaicin test much better, the urge to cough that proceed the cough motor act was detected later, close to C2 concentration. We documented coughs that had not been preceded by the urge to cough at all after nasal menthol pre-treatment in approximately 40% out of tests. Such coughs might have very much simple reflex origin without cortically processed urge to cough induced phenomena.

We mentioned possible trigeminal - olfactory interaction in modulation of cough response in our interpretaion of the observed menthol effects. The nature and extent of interactions between trigeminal and olfactory stimulation are poorly understood however TRPM8 and TRPA1 were detected both on trigeminal and olfactory nerve terminals [29]. Exposure of the nose to the malodorants (unpleasant smells) increases the responsiveness of trigeminal nerves, and this mechanism is mediated by paracrine signalling pathway between olfactory and trigeminal nerves [30]. Patients with acquired olfactory loss exhibit reduced trigeminal sensitivity, possibly due to the lack of these interactions. Considering possible modulation of cough by cortical activity induced by odours is a phenomenon that could contribute to the antitussive efficiency of placebo [31]. In addition, the sweet taste of cough syrups or honey may have modulatory roles through oral/oropharyngeal receptors.

However, the specific role of particular channels in any biological process can only be conclusively proven by the use of selective channel antagonists, which at the moment, are not available for human use in clinical conditions [32]. The results we have obtained in our studies lead us to surmize that the TRPA1 agonist isocyanate significantly modulates urge to cough probably via additional airway irritation instead of capsaicin test itself. Both TRPA1 agonists failed to modulate cough threshold and total cough response at these concentrations, suggesting that higher concentration or repeated exposure to them are necessary to modulate cough response. Both menthol isomers after nasal administration significantly modulated urge to cough, cough threshold, and total cough response probably via the reduction of airway irritation induced during capsaicin challenge. In addition to trigeminal afferents expressing TRP channels, olfactory nerve endings, trigemino – olfactoric relationships, smell perception process and other supramedullar influences should be considered as having potential to modulate the cough response in humans [33].

## Abbreviations

TRPA1: Transient receptor potential channel A1 member; TRPM8: Transient receptor potential channel M8 member; TRPV1: Transient receptor potential channel V1 member; VAS: Visual analogue scale; AITC: Allyl-iso-thiocyanate; C2: Concentration of capsaicin inducing 2 or more coughs; C5: Concentration of capsaicin inducing 5 or more coughs; Cu: Concentration of capsaicin inducing subjective feeling for urge to cough.

## Competing interests

The authors declare no conflict of interests.

## Authors’ contributions

ZB, TB, MB and JP performed nasal challenges, and cough challenges in human volunteers. SG (biomedical engineer), IP & TB analyzed the data, MS considerably edited the manuscript and brought interesting ideas to discussion. VC performed ENT examination and confirmed that subjects meet inclusion criteria to participate in a study. JP wrote the manuscript and she is a leader of this multidisciplinary team. All authors made proof read of the manuscript and meet the criteria for authorship. All authors read and approved the final manuscript.
